# A New Open-Loop Fiber Optic Gyro Error Compensation Method Based on Angular Velocity Error Modeling

**DOI:** 10.3390/s150304899

**Published:** 2015-02-27

**Authors:** Yanshun Zhang, Yajing Guo, Chunyu Li, Yixin Wang, Zhanqing Wang

**Affiliations:** 1 School of Instrumentation and Optoelectronic Engineering, Beihang University, Beijing 100191, China; E-Mails: zhangyanshun@buaa.edu.cn (Y.Z.); spring046400@126.com (C.L.); jinsique-951@163.com (Y.W.); 2 School of Automation, Beijing Institute of Technology, Beijing 100081, China; E-Mail: bitwangzhanqing@163.com

**Keywords:** open-loop fiber optic gyro, angular velocity error, RBF neural network

## Abstract

With the open-loop fiber optic gyro (OFOG) model, output voltage and angular velocity can effectively compensate OFOG errors. However, the model cannot reflect the characteristics of OFOG errors well when it comes to pretty large dynamic angular velocities. This paper puts forward a modeling scheme with OFOG output voltage u and temperature T as the input variables and angular velocity error Δω as the output variable. Firstly, the angular velocity error Δω is extracted from OFOG output signals, and then the output voltage u, temperature T and angular velocity error Δω are used as the learning samples to train a Radial-Basis-Function (RBF) neural network model. Then the nonlinear mapping model over T, u and Δω is established and thus Δω can be calculated automatically to compensate OFOG errors according to T and u. The results of the experiments show that the established model can be used to compensate the nonlinear OFOG errors. The maximum, the minimum and the mean square error of OFOG angular velocity are decreased by 97.0%, 97.1% and 96.5% relative to their initial values, respectively. Compared with the direct modeling of gyro angular velocity, which we researched before, the experimental results of the compensating method proposed in this paper are further reduced by 1.6%, 1.4% and 1.2%, respectively, so the performance of this method is better than that of the direct modeling for gyro angular velocity.

## 1. Introduction

Acting as the angular rate sensor, the fiber optic gyro (FOG) is widely applied in navigation and weapon systems [1,2,3,4]. The nonlinearity of scale factors of the close-loop fiber optic gyro (CFOG) is limited using the homodyne detection technology, while the drift of CFOG is the main error which has an impact on its performance [5,6,7]. The open-loop fiber optic gyro (OFOG), with its minimization structure and open loop detection scheme, is an inertial measurement sensor with middle or low precision. OFOG has many advantages such as small size, low cost and fast response [3,4], leading to a wide range of applications in low precision weapons production, navigational systems for ships, inertial gyro-stabilized platforms and servo tracking systems [8,9,10,11]. Under the comprehensive effects of temperature and angular velocity, the OFOG drift is relatively small and the nonlinearity of scale factors is the main error that restricts the accuracy of its applications [12,13]. Researchers have worked extensively on the modeling and compensation of gyro output [14,15,16,17,18], improving the performance of OFOGs to a certain degree. In view of the nonlinear scale factor of OFOG, [13,19] presented the look-up table and piecewise compensation methods to compensate the OFOG angular velocity error. In [20,21] an OFOG digital demodulation method to reduce the OFOG linearization error was proposed. As for OFOG temperature errors, [22] analyzed the temperature error of Andew Corp’s OFOG and tested the changed data of OFOG bias in the whole temperature range. Considering the comprehensive effects of temperature and angular velocity, [23] took VG951 to establish the gyro output signal model using the polynomial fitting method. In view of the gyro temperature model, [24] proposed the linear regression algorithm and wavelet network algorithm, which described the temperature characteristics of the gyro very well. In [25] the establishment and identification of the nonlinear mixed models of temperature and calibration factor for the compensation of OFOG was accomplished. All the works above involved direct modeling for gyro angular velocity. When the angular velocity is very large, the models’ sensitivity to the angular velocity error is not enough, impacting on the compensation precision of the gyro error. To deal with the problem, this paper expands upon the studies in [3], putting forward a modeling scheme with OFOG output voltage u and temperature T as the input variables and angular velocity error Δω as the output variable. Firstly the angular velocity error Δω is extracted from the OFOG output signals, and then output voltage u, temperature T and angular velocity error Δω are used as the learning samples to train a Radial-Basis-Function (RBF) neural network model. Then the nonlinear mapping model involving T, u and Δω is established. In this way Δω can be calculated automatically to realize the compensation of OFOG errors according to T and u. The model put forward in this paper is validated and tested experimentally, and the results are analyzed in comparison with those in [3].

## 2. The OFOG Angular Velocity Error Model 

### 2.1. The OFOG Composition

**Figure 1:**
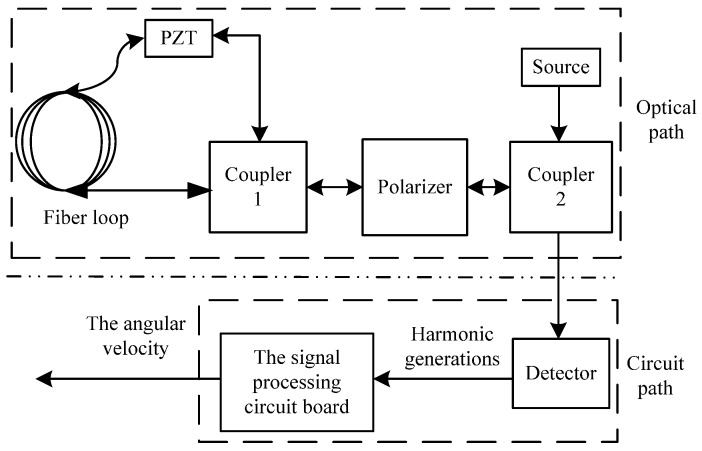
The OFOG composition.

The OFOG consists of two parts: optical path and the signal processing circuit, which are shown in Figure 1. The components of the optical path are source, coupler 2, polarizer, coupler 1, fiber loop and phase modulator (PZT). The optical path is used to measure the angular velocity and produce a corresponding light-intensity signal. Then the light-intensity signal is detected by the signal processing circuit, and is transported into voltage signal corresponding to the angular velocity [26,27,28,29,30].

### 2.2. The OFOG Output Signal 

The output signal of an OFOG contains many harmonics. According to the signal processing circuit board, detection of different harmonic components are various [26]. Among these, detection of the first harmonic is adopted by OFOG because of its simple circuit, high reliability and easy implementation. Its output voltage u can be expressed as:

(1) $$u=u_{0}+k_{d}\sin(k_{g}\text{ω})$$

where $u_{0}$ is the zero-bias voltage of OFOG corresponding to the constant drift of the gyro, and ω is the input angular velocity of OFOG, $k_{d}$ is the scale factor of the circuit, $k_{g}$ is the scale factor of the optical part. In the Equation (1), the relationship between the output voltage and angular velocity is nonlinear, leading to circuit complexity and bad real-time ability. Generally, Equation (1) is simplified by linearization of the sinusoidal function and nominal angular velocity ${\text{ω}}_{c}$ is obtained [3]:

(2) $${\text{ω}}_{c}=(u-u_{0})/(k_{d}\cdot k_{g})=(u-u_{0})/K_{0}$$

where $K_{0}=k_{d}\cdot k_{g}$ is the gyro scale factor calibrated under a certain circumstance before leaving the factory, the so-called nominal scale factor (Fizoptika Corporation calibrates it on the basis of ±10deg/s). As is seen from Equations (1) and (2), there is an angular velocity error Δω between the nominal angular velocity ${\text{ω}}_{c}$ calculated by using $K_{0}$ and the true value of angular velocity ${\text{ω}}_{z}$ [3]:

(3) $$\Delta \omega =\omega_{z}-\omega_{c}$$

Moreover, under the influence of temperature, $u_{0}$ and $K_{0}$ can also lead to gyro angular velocity error. Therefore, under the coactions of angular velocity and temperature, it is hard to conduct modeling and compensation of Δω by analytic method precisely due to its complex structure.

## 3. The Scheme of Modeling and Compensation of OFOG Angular Velocity Error

### 3.1. The Variable Selection for Modeling

The gyro output voltage u and temperature T are the main influencing factors of the gyro angular velocity error. Therefore, u and T are chosen as the input variables for modeling. It is simple and practical with clear physical significance to adopt gyro output voltage u and temperature T as the input variables of the model.

In this paper, the nonlinearity of scale factor, influenced by angular velocity and temperature, is considered as the main error of OFOG. So the scale factor $K({\text{ω}}_{z},T)$ is expressed as a multinomial function involving both temperature and angular velocity during the derivation process to realize the compensation of gyro angular velocity error using the fixed scale factor [11,24]:

(4) $$K({\text{ω}}_{z},T)=K_{0}k_{\omega_{z}}k_{T}$$

where $k_{\omega_{z}}$ is the fixed scale factor relative to the angular velocity, and $k_{T}$ is the fixed scale factor relative to the temperature. After the introduction of the fixed scale factor, the true value of angular velocity ${\text{ω}}_{z}$ is obtained:

(5) $${\text{ω}}_{z}=(u-u_{0})/K({\text{ω}}_{z},T)$$

That is:

(6) $$u=K\left({\text{ω}}_{z},T\right)\times {\text{ω}}_{z}+u_{0}$$

As is seen from Equations (2), (3) and (5), the angular error Δω can be expressed as:

(7) $$\begin{array}{l} \Delta \omega =\omega_{z}-\omega_{c}=(u-u_{0})/K\left(\omega_{z},T\right)-(u-u_{0})/K_{0}\\ =(u-u_{0})[1/K\left(\omega_{z},T\right)-1/K_{0}] \end{array}$$

That is:

(8) $$u=[\frac{1}{1-K\left(\omega_{z},T\right)/K_{0}}]K\left(\omega_{z},T\right)\times \Delta \omega +u_{0}$$

**Figure 2:**
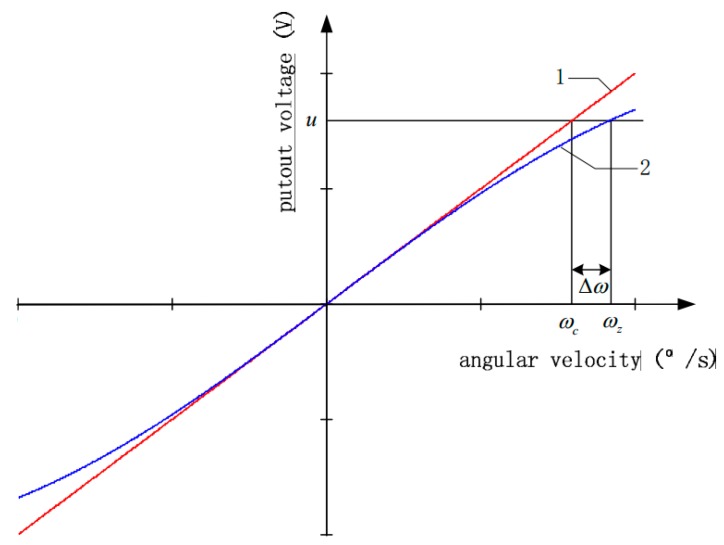
The relationship over u, ${\text{ω}}_{z}$, ${\text{ω}}_{c}$ and Δω.

As $\left|\frac{K\left(\omega_{z},T\right)}{1-K\left(\omega_{z},T\right)/K_{0}}|>|K\left(\omega_{z},T\right)\right|$, it is clear that the relative changing rate of OFOG output voltage u corresponding to the angular velocity error Δω is bigger than that corresponding to the real angular velocity ${\text{ω}}_{z}$. The relationship involving u, ${\text{ω}}_{z}$, ${\text{ω}}_{c}$ and Δω is shown in Figure 2. The line 1 denotes the relationship between u and ${\text{ω}}_{c}$, and the line 2 denotes the relationship between u and ${\text{ω}}_{z}$.

It can be found that the relative change rate of the OFOG output voltage u corresponding to the angular velocity error Δω is bigger than that corresponding to the real angular velocity ${\text{ω}}_{z}$ in Figure 2. Therefore, relative to the direct modeling for gyro angular velocity in [3], the angular velocity error Δω is chosen as the output of the model to improve its sensitivity, which is helpful to reflect the characteristics of Δω because of its greater proportion coefficient between the independent variables and the dependent variables. Therefore, Δω is chosen as the output of the model. In addition, this paper uses the experimental data to analyze the relative change rate of *u*, corresponding to Δω and ${\text{ω}}_{z}$ respectively, and the specific analysis of the result is shown in Section 4.3, which validates the accuracy of the above conclusion.

### 3.2. The Choice and Establishment of the Model

Equation (9) shows the nonlinear relationship over the angular velocity error Δω, gyro output voltage u and temperature T:

(9) Δω=f(u,T)

Therefore, an effective method is needed to set up the mapping model between the gyro output voltage u and the angular velocity error Δω, under the combined effects of temperature T and angular velocity. The neural network method helps to realize the complex nonlinear mapping relationship of input and output via studying the learning samples, according to experimental data. The RBF neural network, with good its capability and global best solution-approaching performance as well as being a fast and easy learned method, does not have the problem of local optima [31,32,33]. 

**Figure 3:**
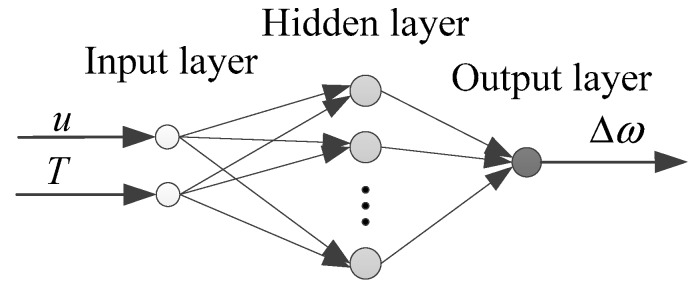
The model of RBF neural network.

To realize the estimation and compensation of the angular velocity error Δω, according to Equation (9), the model of RBF neural network involving the gyro output voltage u, temperature T and the angular velocity error Δω is established through studying experimental data for modeling. As is shown in Figure 3, u and T act as the inputs and Δω as the output in the model of RBF neural network.

### 3.3. The Method of Error Compensation

**Figure 4:**
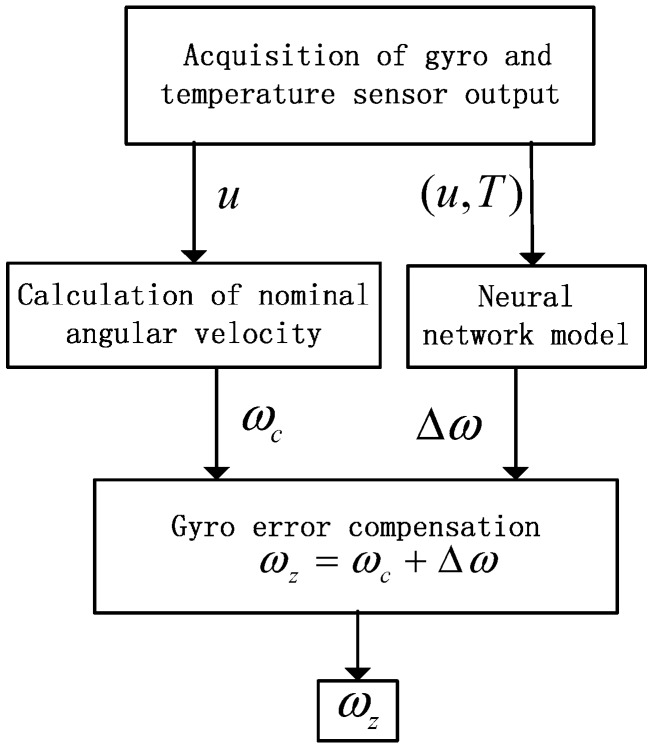
The flow of gyro angular velocity error compensation.

The flow of the gyro angular velocity error compensation is shown in Figure 4: (1) the gyro output voltage u and temperature T are collected; (2) we make use of Equation (2) to calculate the nominal angular velocity; (3) the gyro output voltage u and temperature T are sent to the RBF neural network, which figures out the angular velocity error Δω; (4) Equation (3) is utilized to calculate the angular velocity ${\text{ω}}_{z}$ and estimate the real value of angular velocity precisely. The experimental data collected in this article include the output change of OFOG which is caused by changes in drift and scale factor and can be expressed as the change of angular velocity error Δω. Therefore the model proposed in this paper contains the error of the drift and scale factor nonlinearity of OFOG related to temperature, and the proposed method corresponding to the model can compensate the error in the whole temperature range.

This paper chooses a RBF neutral network model as the modeling method which adopts OFOG output voltage u and temperature T as the input variables and angular velocity error Δω as the output variable. In addition, this paper uses the experimental data for learning, validation and precision analysis of RBF neural network model. In order to ensure credibility of the method and conclusion, one part of the collected data are used for the RBF neural network training, and the others are utilized for model validation.

## 4. Experiments and Results

### 4.1. Experimental Scenario

**Figure 5:**
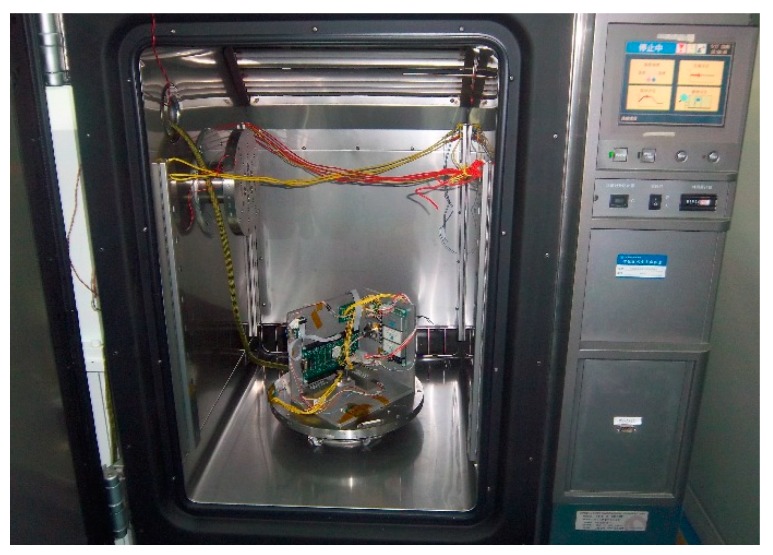
The photo of experimental setup.

VG095M, an OFOG produced by the Russian Fizoptika Corporation (Moscow, Russia), is chosen as the experimental component. In this paper, further study is done based on the previous researches in [3]. The experimental equipment and the data acquisition and processing system are the same as before. So, detailed descriptions are omitted here. The photo of experimental setup is shown in Figure 5.

Firstly, the data acquisition scheme is designed for different temperatures and angular velocities. Then the experimental data, collected according to the data acquisition scheme, are divided into modeling data and verification data. Secondly, the modeling data are used as the learning samples of the neural network introduced in Section 3.2. Finally, the verification data are used to verify the error compensation method introduced in Section 3.3.

### 4.2. Data Acquisition

First of all, with the turntable controlled by a thermostat, the output voltage of the VGO95M is acquired under the influences of different temperatures and angular velocities. Secondly, the nominal angular velocity ${\text{ω}}_{c}$ is calculated using Equation (2). Then Equation (3) is used to extract the angular velocity error Δω. To establish the model of angular velocity dynamic error of the VG095M, the collected data for modeling, namely the gyro output voltage u, temperature T and the angular velocity error Δω, are used as the learning samples of the neural network. Finally, experimental data different from the modeling data are acquired to verify this model.

Set the temperature of thermostat to (−30+10*i)℃ (i=0,1⋯7,8) respectively. When the temperature is stabilized, different ${\text{ω}}_{z}$ are inputted into the rotary table. The angular velocity ±300deg/s, ±250deg/s, ±200deg/s, ±150deg/s, ±100deg/s, ±60deg/s, ±10deg/s, 0deg/s are for modeling and ±280deg/s, ±180deg/s, ±120deg/s, ±40deg/s, ±20deg/s, ±15deg/s, ±8deg/s, ±5deg/s, ±1deg/s are for model verification. The gyro output information is acquired at different test points. Δω is extracted from experimental data by solving Equations (2) and (3), which is utilized to establish RBF neural network model.

### 4.3. The Analysis of Relative Change Rate

**Figure 6:**
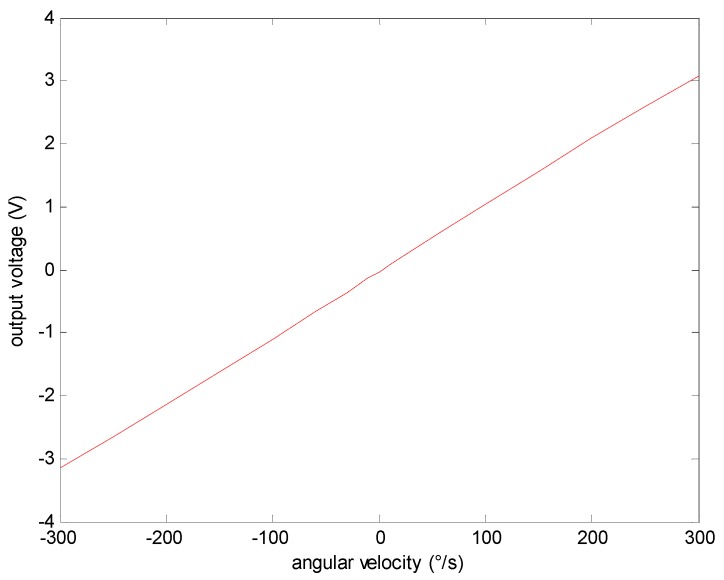
The relationship between output voltage and angular velocity.

**Figure 7:**
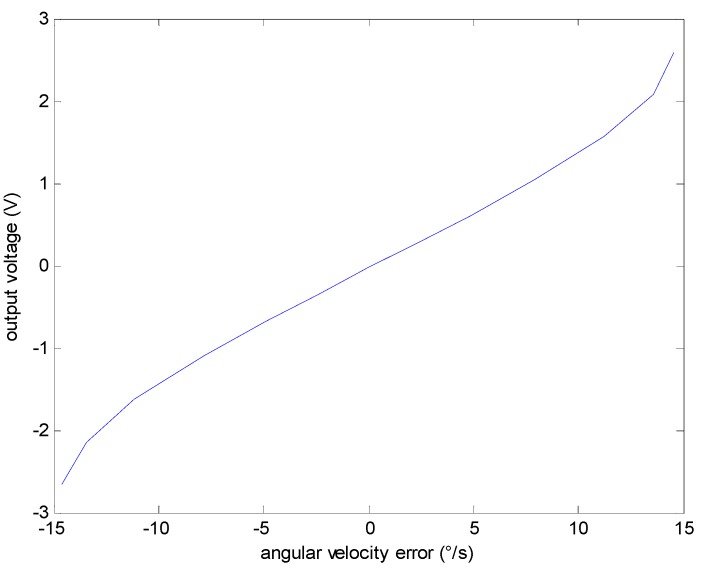
The relationship between output voltage and angular velocity error.

The output voltage of gyro can be expressed as the function of the angular velocity ${\text{ω}}_{z}$ according to Equation (6) or the angular velocity error Δω according to Equation (8). The mapping relationship is similar under different temperatures. The analysis of experimental data in 20 °C is shown in Figure 6 and Figure 7, respectively. This article validates the above conclusion through concrete analysis of the experimental data.

Figure 6 and Figure 7 shows that the relative changing rate of OFOG output voltage u corresponding to the angular velocity error Δω is faster (almost 20 times) than that corresponding to the real angular velocity, so, the angular velocity error Δω is chosen as the output of the model rather than the angular velocity to improve the model’s sensitivity. Also it is better to compensate the angular velocity error.

### 4.4. The Analysis of the Angular Velocity Error

**Figure 8:**
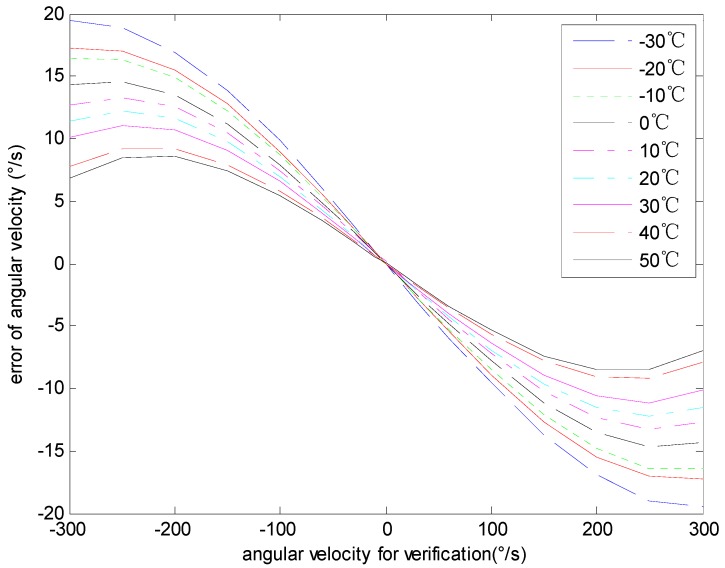
The angular velocity error of modeling data.

**Figure 9:**
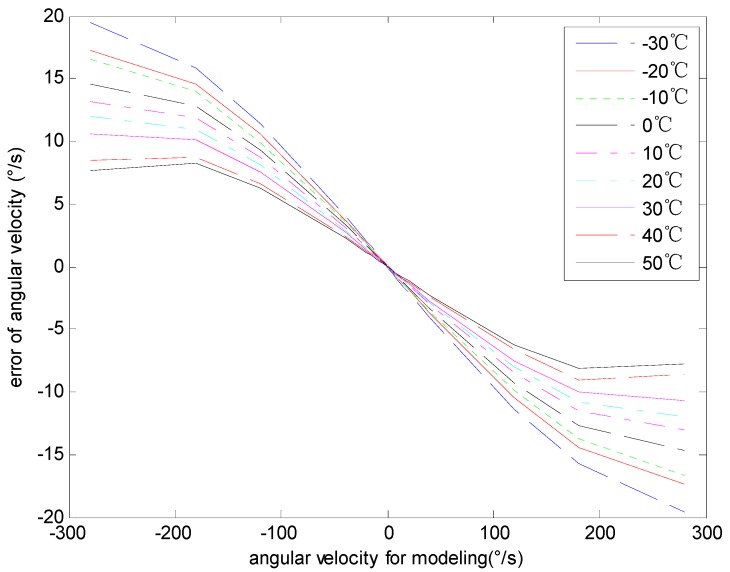
The angular velocity error of model verification data.

The experimental data acquired in Section 4.2 are processed to analyze the characteristics of the angular velocity error. The data for modeling and verification are shown in Figure 8 and Figure 9 respectively. Figure 8 shows the relationship between the input angular velocity for modeling and the angular velocity error with different temperatures. Figure 9 shows the relationship between the input angular velocity for verification and the angular velocity error with different temperatures. In Figure 8 and Figure 9, the curves from top to bottom respectively represent different temperatures from 30 °C to 50 °C.

The Figure 8 shows that the same angular velocity corresponds to different errors with different temperatures. Moreover, the relationship among them is nonlinear. The maximum angular velocity error is 19.46deg/s, while the minimum error is −19.53deg/s and the mean square error is 9.34deg/s.

The Figure 9 shows that the performance and the rule of the angular velocity error for verification or modeling are alike. The maximum angular velocity error of model verification data is 19.47deg/s, while the minimum error is −19.56deg/s and the mean square error is 6.73deg/s. As is shown in Figure 8 and Figure 9, the gyro angular velocity error caused by temperature and input angular velocity are coupled. In the whole testing scope, the gyro angular velocity error is an irregular curve, and it is hard to express with an analytical formula, whereas the testing data analysis result demonstrates that the angular velocity error is highly repeatable and is capable for modeling and compensating the errors by establishing a RBF neural network model.

### 4.5. The Neutral Network Modeling

**Figure 10:**
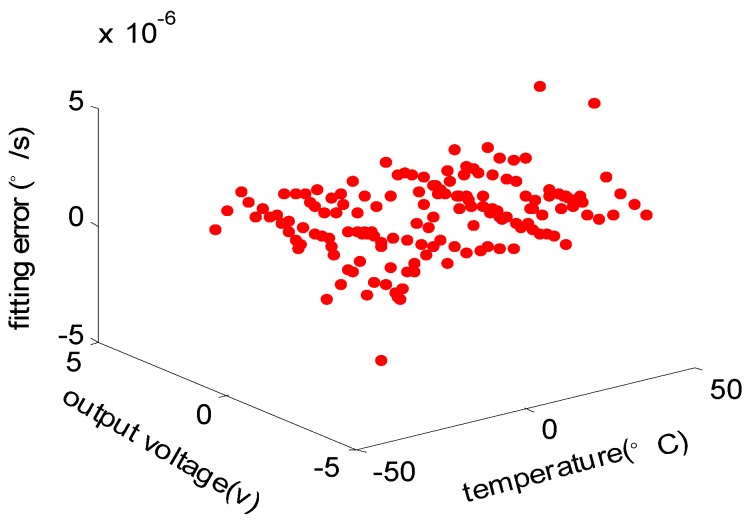
The fitting error of RBF neural network model.

The data collected for modeling, namely the gyro output voltage u, temperature T and the angular velocity error Δω, are used as the learning samples of the neural network introduced in Section 3.2. Through studying the learning samples, the model of RBF neural network over the gyro output voltage u, temperature T and the angular velocity error Δω is established according to the Equation (9). The training error of neural network is selected as $5\times 10^{-6}$, and dispersion coefficient is 0.96. The fitting error of training RBF neural network is shown in Figure 10.

The maximum fitting error is $\text{4.7558}\times 10^{-6}\text{deg}/s$, while the minimum fitting error is −3.4265 × 10^−6^ deg/s and the mean square error is $\text{7.5826}\times 10^{-7}\text{deg}/s$. Therefore, it’s obvious that the RBF neural network model built in the paper reflects the mapping relationship over gyro output voltage, temperature and the angular velocity error Δω, which can estimate Δω with high precision according to gyro output voltage u and temperature T.

### 4.6. The Analysis of the Effect of Error Estimation

**Figure 11:**
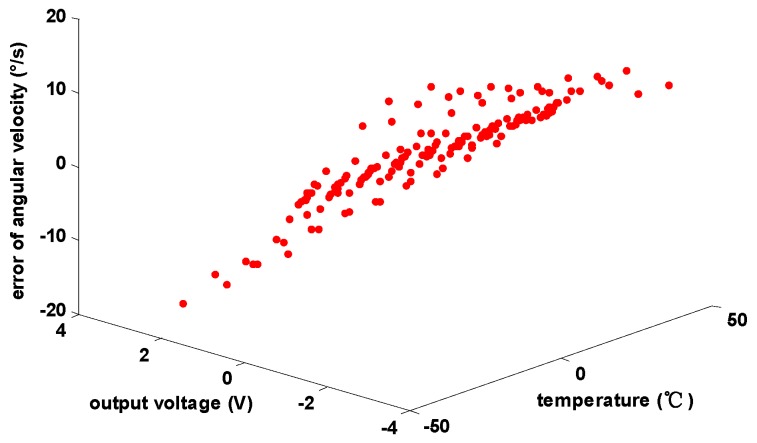
The angular velocity error of test points.

The data for verification are sent to the RBF neural network to figure out the angular velocity error Δω, which is shown in Figure 11.

**Figure 12:**
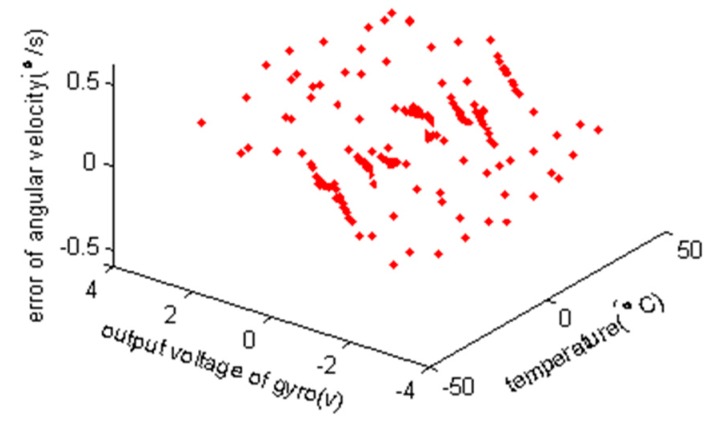
The angular velocity error of the verification data after compensation.

The sum of the angular velocity error Δω and the nominal angular velocity ${\text{ω}}_{c}$ calculated by Equation (2) is the estimation of angular velocity. Then, the gyro angular velocity error of test points can be calculated by comparing the estimation of angular velocity with the true value of the angular velocity, as shown in Figure 12.

After compensation, the maximum angular velocity error of model verification data is 0.59deg/s, while the minimum error is −0.57deg/s and the mean square error is 0.23deg/s. The result shows that the RBF neural network model established in the paper has good generalization ability, and can realize the compensation of gyro errors with high precision in the whole temperature scope of the gyro. The maximum, the minimum and the mean square error of gyro angular velocity are decreased by 97.0%, 97.1% and 96.5% relative to their initial values, respectively. In [3] the gyro angular velocity was directly taken for modeling using the same data, and the maximum, the minimum and the mean square error of gyro angular velocity were also reduced by 95.4%, 95.7% and 95.3%, respectively, after compensation by that model. Also compared with the direct modeling for gyro angular velocity [3], the errors of the compensating method proposed in this paper are further reduced by 1.6%, 1.4% and 1.2%, respectively.

## 5. Conclusions

According to the characteristics of the OFOG angular velocity error, the signal of the angular velocity error Δω is extracted from the OFOG output signal. Meanwhile, the modeling and compensation method of the angular velocity error of OFOG, based on a RBF neural network, is proposed. The RBF neural network can be used to directly calculate the angular velocity error. Then the angular velocity can be estimated together with the nominal angular velocity. The result of the processing experimental data shows that the RBF neural network model built in the paper reflects the mapping relationship over output voltage *u* , temperature T and the angular velocity error Δω, rendering the estimation and compensation of the dynamic angular velocity error with high precision, high sensitivity and global best approaching performance in the whole temperature scope possible. It is clear that the performance of the method proposed in this paper is better than that of the direct modeling of gyro angular velocity.

## References

[1] Shen C., Chen X. (2012). Analysis and modeling for fiber-optic gyroscope scale factor based on environment temperature. Appl. Opt..

[2] Chen X., Song R., Shen C., Zhang H. (2014). Application of a genetic algorithm Elman network in temperature drift modeling for a fiber-optic gyroscope. Appl. Opt..

[3] Zhang Y.S., Wang Y.Y., Yang T., Yin R., Fang J.C. (2012). Dynamic angular velocity modeling and error compensation of one-fiber fiber optic gyroscope (OFFOG) in the whole temperature range. Meas. Sci. Technol..

[4] Zhang Y.-S., Cheng J.-B., Tang J.-Q. (2004). Key technology and application prospect of the one-fiber fiber-optical gyroscope. Opt. Tech..

[5] Chen X., Shen C. (2012). Study on error calibration of fiber optic gyroscope under intense ambient temperature variation. Appl. Opt..

[6] Chen X., Shen C. (2013). Study on temperature error processing technique for fiber optic gyroscope. Opt. Int. J. Light Electron Opt..

[7] Song R., Chen X., Shen C., Zhang H. (2014). Modeling FOG Drift Using Back-Propagation Neural Network Optimized by Artificial Fish Swarm Algorithm. J. Sens..

[8] Emge S., Bennett S., Dyott R., Brunner J., Allen D. (1997). Reduced minimum configuration fiber optic gyro for land navigation applications. IEEE Aerosp. Electron. Syst. Mag..

[9] Dyott R.B., Bennett S.M., Allen D., Brunner J. Development and Commercialization of Open Loop Fiber Gyros at KVH Industries (Formerly at Andrew). Proceedings of the Optical Fiber Sensors Conference Technical Digest.

[10] Moeller R.P., Burns W.K., Frigo N.J. (1989). Open-loop output and scale factor stability in a fiber-optic gyroscope. J. Lightw. Technol..

[11] Thielman L.O., Bennett S., Barker C.H., Ash M.E. Proposed IEEE Coriolis Vibratory Gyro standard and other inertial sensor standards. Proceedings of the Position Location and Navigation Symposium.

[12] Liaw C.-Y., Zhou Y., Lam Y.-L. (1998). Characterization of an open-loop interferometric fiber-optic gyroscope with the Sagnac coil closed by an erbium-doped fiber amplifier. J. Lightw. Technol..

[13] Medjadba H., si Mohamed L.M. (2006). Low cost technique for improving open loop fiber optic gyroscope scale factor linearity. Inf. Commun. Technol..

[14] Wang Z., Yang Y., Lu P., Li Y., Zhao D., Peng C., Zhang Z., Li Z. (2014). All-Depolarized Interferometric Fiber-Optic Gyroscope Based on Optical Compensation. IEEE Photon. J..

[15] Ferreira E.C., de Melo F.F., Siqueira Dias J.A. (2007). Precision analog demodulation technique for open-loop Sagnac fiber optic gyroscopes. Rev. Sci. Instrum..

[16] Almeida V.R., da Silva A.C., Oliveira J.EB. High Dynamic Range Fiber Optic Gyroscope Demodulation Technique Based on Triangular Waveform Phase Modulation. Proceedings of the International Microwave and Optoelectronics Conference, 1999.

[17] Avanaki M.R.N. Full Progress of Digital Signal Processing in Open Loop-IFOG. Proceedings of the Optical Fiber Communication & Optoelectronic Exposition & Conference (AOE 2006).

[18] Rodriguez R.B.G., Ferreira E.C. Zero-Crossing Demodulation for Open Loop Fiber Optic Gyroscopes. Proceedings of the 2001 International Microwave and Optoelectronics Conference.

[19] Zhang Y., Zhou Z., Fan J. (2008). Error compensation method based on neural network for open-loop FOG. J. Data Acquis. Process..

[20] Jin Z., Yang Z., Ma H., Ying D. (2007). Open-Loop Experiments in a Resonator Fiber-Optic Gyro Using Digital Triangle Wave Phase Modulation. IEEE Photon. Technol. Lett..

[21] Hotate K., Harumoto M. (1997). Resonator fiber optic gyro using digital serrodyne modulation. J. Lightw. Technol..

[22] Bennett S.M., Emge S., Dyott R.B. Fiber Optic Gyroscopes for Vehicular Use. Proceedings of the IEEE Conference on Intelligent Transportation System.

[23] Wang X., Ma S. (2009). Nonlinearity of temperature and scale factor modeling and compensating of FOG. J. Beijing Univ. Aeronaut. Astronaut..

[24] Zhang G., Deng Z., Fu Z.-X. (2003). Temperature Modeling Study for Gyroscope. J. Syst. Simul..

[25] Wang X., Li J., Xu H., Li A. (2007). Research of FOG’s Error Modeling Based on Temperature and Scale Factor Nonlinearity. J. Syst. Simul..

[26] Spammer S.J., Swart P.L. (1993). A quadrature phase tracker for open-loop fiber-optic. IEEE Trans. Circuits Syst..

[27] Wang Q., Yang C., Wang Z. A Novel Digital Signal Processing System for Open-Loop Fiber Optic Gyroscope. Proceedings of the Communications and Photonics Conference (ACP) (2012 Asia).

[28] Park H.G., Ah Lim K., Chin Y.-J., Kim B.-Y. (1997). Feedback effects in erbium-doped fiber amplifier/source for open-loop fiber-optic gyroscope. J. Lightw. Technol..

[29] Terrel M.A., Digonnet M.J.F., Fan S. (2012). Resonant Fiber Optic Gyroscope Using an Air-Core Fiber. J. Lightw. Technol..

[30] Zhou K.J., Hu K.K., Dong F.Z. (2014). Single-mode fiber gyroscope with three depolarizers. OPTIK.

[31] Burse K., Yadav R.N., Shrivastava S.C. (2010). Channel equalization using neural networks. IEEE Trans. Syst. Man Cybern..

[32] Malleswaran M., Vaidehi V., Deborah S.A., Manjula S. (2010). Comparison of RBF and BPN neural networks applied in INS and GPS integration for vehicular navigation. Int. J. Electron. Eng. Res..

[33] Steinwart I., Hush D., Scovel C. (2006). An explicit description of the reproducing kernel Hilbert spaces of Gaussian RBF kernels. IEEE Trans. Inf. Theory.

